# Supplementary material for: Are carbon emissions trading and green financial instruments synergistic? -Comprehensive quantitative research based on content analysis
Source: PLoS One. 2024 Mar 7;19(3):e0298601. doi: 10.1371/journal.pone.0298601 (PMC10919601; doi:10.1371/journal.pone.0298601)
Supplement: S1 Data — (ZIP) [file pone.0298601.s001.zip › Data/地级市Z_F工作报告.zip!/║┌┴·╜¡/╝╤─╛╦╣╩╨/└┤╫╘╣1⁄2╓┌║┼í╛┬φ┐╦╩2╛▌═°í┐_2010.docx]

各位代表：

　　现在，我代表市人民政府向大会报告工作，请予审议，并请各位政协委员和列席会议的同志提出意见。

　　一、2009年工作回顾

　　2009年是极不平凡的一年。我们坚持以科学发展观为统领，在省委、省政府和市委的领导下，深入实施“十项重点工程”，全面推进“两区两带一城”建设，取得了应对金融危机，保增长促发展的决定性胜利，保持了经济社会又好又快的发展态势。主要经济指标增幅超过全省平均水平，提前一年实现了“十一五”规划目标，较好地完成了市十四届人大四次会议确定的各项任务。全市地区生产总值完成432.7亿元，比上年(下同)增长14.1%，增幅居全省第二位，实现五年翻一番；财政一般预算收入完成14.5亿元，增长32.1%，增幅居全省第四位，实现三年翻一番。

　　1、项目投资建设成效显著，重大项目取得新突破。深入实施大项目牵动工程，全市固定资产投资实现170.9亿元，增长51.1%，增幅创历史最高水平，实现五年翻两番。重点项目进展顺利。全市开工建设千万元以上项目274个，增加66个，其中亿元以上项目50个，增加11个。在列入省重点推进的16个项目中，投资21亿元的前进至抚远铁路等13个项目实现开复工建设，投资30亿元的华电60万千瓦扩建等3个项目实现竣工投产。同江港区等18个口岸通道基础设施项目实现开复工建设，完成投资48亿元。产业项目建设步伐加快。新能源实施项目7个，完成投资23.8亿元，桦南长寿山、驿马山风电、郊区猴石山风电、同江街津口风电等4个风电项目新增装机能力20万千瓦，全市风电装机突破60万千瓦，占全省40%，保持全省领先。新材料和新型建材实施项目23个，完成投资9.2亿元，总投资7.5亿元的东兴煤化工年产1.6亿立方米煤气项目竣工投产，焦炭生产能力达到105万吨。3万吨海绵钛项目全面建设；鸿基集团日产2500吨水泥熟料及佳木斯、同江200万吨水泥粉磨站投产达产。农副产品加工实施项目19个，完成投资6.8亿元，其中益海嘉里二期完工，形成了水稻加工能力40万吨；江苏雨润200万头生猪屠宰加工项目竣工，这两个企业已经成为全省同行业中单厂产能最大的企业。先进装备制造业实施项目15个，完成投资5.2亿元，特种电机生产能力达到800万千瓦、掘进机综合生产能力达到400台、大型联合收割机生产能力达到8000台，超高压电缆生产能力达到7千公里。重大项目前期进程加快。投资21亿元的中俄黑龙江铁路大桥、投资430亿元的哈佳城际铁路和投资4.5亿元的抚远机场完成可研上报待批。投资6亿元的佳木斯机场迁建、投资42亿元的悦来航电枢纽启动了前期工作。总投资500亿元的400万千瓦核电项目已与中广核公司签署了合作协议。黑瞎子岛保护与开放开发上升到国家战略，国务院批复建设对俄合作示范区，各项规划和护岸工程、上岛桥等基础设施项目前期工作全面启动。金融运行和招商引资创历史最好水平。金融机构贷款余额达到280.9亿元，增长30.6%。驻佳金融机构全部实现扭亏为盈。建设银行、光大银行与我市分别签署战略合作协议，意向贷款额度达到400亿元。获准筹建的12家小额贷款公司全部开业，数量列全省第二位。招商引资到位资金实现92.3亿元，增长49%，实现五年翻3.5番；引进外资实现5128.6万美元，增长10.1%。中国建材、中煤能源、山东鲁能、龙煤矿业、杭州九阳、葵花药业等一些大企业签约落地。

　　2、工业经济平稳快速增长，质量和效益同步攀升。工业大打保增长攻坚战，全市规模以上工业实现主营业务收入205.7亿元，增长22.7 %，实现增加值46.9亿元，增长18.3%，均实现四年翻一番以上；工业利税总额实现16.9亿元，增长45.9%，实现五年翻两番。优势产业和重点骨干企业支撑作用明显。全市农副产品加工业实现销售收入87亿元，占全部工业的42.3%；装备制造业实现利税9.2亿元，占全部工业的54.8%。37户重点骨干企业发展势头良好，实现主营业务收入占全部工业的65.7%，实现利税占全部工业的60.6%。工业项目形成新的增长点。迪尔佳联年产1400台玉米收割机、沃尔德紫外光辐照交联电缆料等31个项目相继投产，新增销售收入23亿元。民营经济成长迅速。实施“育小扶中抓大”战略，全年民营经济实现营业总收入290亿元，增长32.5%；实现增加值74亿元，增长32.4%，实缴税金8.1亿元，增长27.8%。县域工业发展步伐加快。县域工业实现增加值18.7亿元，增长26.5 %；实现主营业务收入80.8亿元，增长31.8%，占全市工业比重近40%。国企改制取得重要成果。93户改制企业留守处按战线合并为5个，对179户企业改制存在的遗留问题进行梳理解决，完成并轨转制企业15户。东风造纸、饲料公司、联合收割机厂3户企业完成政策性破产，葵花药业收购哈高科佳中药，七彩毯业收购侨龙毛纺。

　　3、现代农业核心区建设快速推进，粮食增产农民增收。以规模化、标准化、水利化、农机化、产业化建设为重点，加快发展现代农业，粮食总产实现95.22亿斤，再创历史新高。农民人均纯收入首次突破6000元大关，增长25.1%，收入水平跃升到全省第四位，增幅列第二位。全力推进粮食产能工程建设。积极倡导和推广复合型规模化生产模式，规模化生产面积达到742万亩，占耕地总面积的39.4%。种植业结构调整效果明显，水稻播种面积增加40万亩。积极推进畜牧业发展。推广普及“五环”养殖技术，各类规模养殖场、大户发展到2071个，养殖小区发展到350个，生猪出栏量达到308万头，增长15.5%；肉蛋奶产量达到47.3万吨，增长20.4%，牧业产值实现50.2亿元，占农业总产值的46.5%。农机建设年深入推进。富锦市争取国家投资1亿元整县推进农机项目。全市组建300万元以上农机合作社18个，购置大中型轮式拖拉机600台，大型配套农机具430台(套)。水利灌区建设步伐加快。8个三江平原灌区项目列入全省千亿斤粮食产能规划，临江、幸福、悦来、引汤等4个灌区全面建设。农业产业化经营实现新突破。全市规模以上龙头企业达到128户，新增18户，订单生产面积达到623万亩。全市水稻加工能力已达200万吨以上，大豆80万吨、玉米50万吨。场县共建深入推进。农场完成跨区作业172万亩，场县共建领域已经从粮食生产、科技示范、农机合作扩大到市场开发、人才共享等33个领域。外向型农业和优质农业快速发展。境外农业开发面积达到74.74万亩，境内对俄果菜基地扩大到9.76万亩。通过国家无公害环评、绿色食品(有机、无公害)标准化生产基地13个、580万亩。新农村建设扎实推进。完成投资4.7亿元，建设农村公路1246.6公里。新增植树造林面积29.1万亩。投入资金1800万元，解决60个村屯、3万人的饮水安全问题，农村自来水入户率达到60%以上。 投资2240万元，完成“一池三改”建设项目3200户。落实良种补贴1.1亿元，购机补贴5080万元，增长1.07倍。土地流转面积完成202.5万亩，新增转移农村劳动力2.9万人，实现劳务总收入19.5亿元，增长11%。

　　4、内贸发展加快，外贸下滑得到遏制。坚持扩大内需稳外需，全市实现社会消费品零售总额178.1亿元，增长20.7%；实现外贸进出口总额20. 32亿美元，下降6.9%，降幅低于全省23个百分点，外贸额实现五年翻两番。城乡市场进一步活跃。深入开展“家电下乡”、“万村千乡市场工程”和“双百市场工程”，大力培育消费热点，全市共销售家电下乡产品4.2万余台。市场体系建设加快，温州批发城、安庆农资市场竣工交付使用，农机大市场、红旗大市场二期、商业大厦改造扩建、秋林公司改造扩建等市场建设进展顺利。外贸下滑势头得到有效遏制。积极推进对俄合作适应性战略调整，全年新增实际经营业绩外贸主体20户；对亚洲、北美、大洋洲进出口均增长2倍以上，加工贸易进出口额2345万美元，增长1.3倍。口岸通道建设步伐加快。同江、抚远口岸被国务院批准实行外国人入境落地签证。佳木斯航空口岸正式通过国家验收，全市对俄水运、陆路、空中立体通道格局初步形成。同江口岸东西港区新增货物吞吐能力20万吨；富锦东平港区开工建设。佳木斯至集贤高速公路建成通车；前进至抚远铁路，集贤至同江高速公路，富锦跨松花江大桥、抚远至黑瞎子岛公路开工建设。投资3800万完成客运枢纽站和中心站改扩建工程。园区建设实现重大突破。佳木斯高新区被省政府批准晋升为省级高新区。省级佳南现代农业科技园区建设取得重要进展，同江在俄下列宁斯阔耶木材加工园区已纳入两国资源合作规划，成为国家级境外园区。各县(市)区辟建的工业园区竞相发展，集聚了一批项目。三江特色旅游带建设进展较快。富锦黑渔泡湿地公园和三江平原湿地宣教馆、汤原亮子河国家森林公园等景区建成开放，敖其湾赫哲族文化旅游区建设加快。成功举办了杏花节、国际旅游节、知青节、国际泼雪节等系列节会。全年共接待国内游客208万人次，实现旅游收入8.6亿元；接待境外游客12.3万人次，创汇2214万美元。

　　5、城市改造力度加大，城市功能显著提升。以十二届省运会筹备为契机，全面推进城市9项重点工程建设，是近年来投资最大、项目最多、进展最快的一年。城市规划取得新成果。相继编制完成了滨水城市总体规划和体育会展中心、体育场等123项重点工程规划。体育场馆及路桥建设如期推进。场馆建设高速度、高质量推进，完成投资1.1亿元，主体工程基本结束。改造和新建长安、友谊、中山、和平等19条主要路段，维修红旗、站前、保卫等33条街道和45条巷道，新建道路面积18.9万平方米，红旗桥、安庆南桥、中山桥全部竣工通车。长安路人防续建工程投入使用。“国家园林城”创建深入开展。重点推进园林绿化与改造工程，友谊、红旗、长安3条精品路主体绿化工程全面完成；杏林湖等4个公园完成提档升级；双拥、西浦两个新建公园建成开园；栽植各类树木316.5万株，完成绿化面积46.8万平方米。城市管网建设取得阶段性成果。天然气新用户安装2.5万户；热网改造5公里，并网小锅炉23座、50万平方米，新增供热面积206万平方米，集中供热普及率达到70%。“一棚一草”建设取得新突破。完成投资29.6亿元，棚户区改造项目38个、120万平方米，相当于前4年的总和，旧小区改造完成5.3万平方米，新建经济适用住房19.1万平方米。配建实物配租廉租房300户，新增租金补贴6538户，享受廉租房政策总户数达13337户。拆除泥草房8939户、60.36万平方米，建成砖瓦房10116户、84.84万平方米，被省政府评为全省泥草房改造先进市。城市经营水平不断提升。全年供地项目35宗，2119.54公顷，本级土地出让金收缴2.93亿元。住房公积金当年归集额突破4亿元，增长21.2%，贷款发放突破3亿元，增长67%。利用开发银行贷款4亿元进行棚户区改造，启动新时代城投公司10亿元城投债券发行工作。房地产市场进一步规范，对历年累积的104个项目进行清理整顿，追缴税费6574万元，3600户产权证照问题得到解决。市容环境进一步改善。城市公交体制改革取得突破，成立佳运公交公司，253台城区中巴车下线停运，改善了市民的出行环境。提高“三清”作业标准，投资500万元的垃圾处理场渗沥液处理工程建设完成，市区生活垃圾实现日产日清，重点区域的脏乱差问题得到集中整治。列入国家和省规划的14个松花江流域水污染防治项目，11个已投入使用，其余3个基本建成。饮用水源地水质达标率达到100%；二级以上良好天气达到336天，增加5天。全市万元GDP能耗下降5.24%，化学需氧量和二氧化硫排放量消减6700吨、12000吨。我市成为获得国际能源总署“全球区域能源气候贡献奖”的唯一亚洲城市。

　　6、民生工程扎实推进，城乡居民得到更多实惠。抢抓国家政策机遇，集中力量推进“十项民生工程”。全力推进扩大就业工程。全市共完成各类培训8.1万人，实现城乡新就业12.8万人，零就业家庭保持动态为零。发放社保补贴1.4亿元，为5030人发放小额担保贷款1.1亿元。全市城镇登记失业率控制在4.3%，低于省控制指标0.2个百分点。不断提高社会保障水平。城镇社会养老保险、失业保险、医疗保险参保人数分别达到22.9万人、18.9万人和65.1万人。全市参加新农合农民98.4万人，参合率95.52%。市区所属乡镇及富锦市列入全国新型农村社会养老保险试点，有3.4万农民享受新养老保险的优惠政策；“五七工”、“家属工”纳入城镇社会养老保险省级统筹1.7万人。社会救助力度进一步加大。市辖区低保标准由每月194元提高到221元，城市低保户实现5.1万户，12.5万人；农村低保标准由每年900元提高到1200元，农村低保户实现4.3万户，11.2万人。市本级综合救助达247.9万人次，金额1.92亿元。社会福利基础设施不断完善。社会福利大厦建设如期竣工；老年公寓投入使用；精神病院二期项目进入收尾阶段；儿救中心建设项目列入省级建设规划；东风社会福利服务中心项目动工兴建。科技文化医疗教育事业稳步发展。启动建设各类科技创新平台30个，实施省级以上科技创新项目50个，高新技术产值增长25%。公共文化服务体系建设顺利推进，图书馆新馆开工建设。疾控水平进一步提高，成功遏制“甲流”爆发势头。中医院综合楼、肛肠医院综合楼竣工投入使用。职业技术学院实训基地和旅游大厦开工建设。义务教育标准化学校建设取得突破，26所学校达到标准化条件。教育公平取得新进展，落实农村义务教育学校经费补助资金4722.3万元，全面落实义务教育免杂费制度，有6万名学生享受到了政策恩惠；有11047名中等职业学校和普通高中品学兼优的贫困生得到助学补助；6035名农民工子女就近入读我市公办中小学校，与城市职工家庭子女享受同等待遇。佳木斯一中蝉联全省高考状元五连冠。平安佳木斯建设迈上新台阶。社会治安形势持续平稳好转，成为全省首批平安市，8个县(市)区成为省级“平安县(市)区”，桦川县被命名为“全国社会治安综合治理先进县”。组织开展疑难重点信访案件百日攻坚战和信访积案化解攻坚战，筹措资金3315万元，573起多年积累的疑难信访案件得到解决，积案化解取得历史性突破。安全生产四项主要控制指标全面下降，被评为全省安全生产优秀地市。

　　7、政府自身建设进一步加强，执政能力不断提升。学习实践科学发展活动取得显著成效，政府的执行力和推动发展的能力进一步增强。自觉接受人大的依法监督、政协的民主监督和公民、社会、媒体的监督，对140件人大代表建议、228件政协委员提案办复率达100%。行政审批效能和水平大幅提升，“一整合、两到位、四压缩”全面落实，受理各类审批事项74807件，按时办结率100%，接待群众投诉咨询125件，办结率100%，群众满意率达98%以上。全面建立政府信息公开机制，阳光政府建设迈出新步伐。政府应急管理工作全面推进，多起突发公共事件得到及时处置。规范权力运行，发展环境进一步优化。

　　各位代表！过去的一年，我们经受住了前所未有的考验，取得了来之不易的成绩。全市上下在困难和挑战面前，所展现的自强不息的精神风貌、高昂饱满的工作热情、科学扎实的工作态度和卓有成效的工作成果，必将成为推动佳木斯未来更好更快发展的强大力量。一年来，我们面对危机见事早，把握了发展的主动权，应对危机信心足，坚定发展的决心不动摇。我们连续多次召开专题会议分析研判形势，切实做到审时度势、未雨绸缪。在应对“危”上，果断提出要提早谋划，制定对策，把困难估计足，同时坚定发展的信心。在把握“机”上，抢抓国家扩大内需政策所带来的重大机遇，早做准备，主动出击，使化不利为有利，化危机为机遇成为全市上下的广泛共识。一年来，我们立足当前，着眼长远，明晰工作思路，准确把握经济发展的走向和脉络。市委确定的“两区两带一城”发展战略，规划了佳木斯科学跨越发展的远景方向；以“十项重点工程”为载体，以“三保一推进”为内涵的发展思路，实现了与国家宏观政策和省发展战略的有效对接。市政府把“保增长、促发展”作为经济工作的主题，制定了一系列高于全省平均水平、自加压力的发展目标，确定了把投资作为主要拉动力量、工业作为主攻方向、农业作为坚实基础、内外贸作为重要推动力量、民生工程作为落脚点和增长点的具体应对举措。一年来，我们统筹兼顾，重点推进，以强有力的对策和措施攻坚克难，始终保持发展的强劲态势。围绕国家产业、财政、货币政策的实施，提前规划了“十大投资领域”，总投资额超过4000亿元的重点项目，成为赢得国家政策支持的有效载体和重要依托。实施工业“一保二增三减”的应对措施，出台了帮助企业保增长、促发展若干政策及实施办法十二条，重点企业的110余个生产要素保障等问题得到有效解决。对37户主营业务收入超亿元企业实行重点跟踪监控，进行旬统计、旬调度，保持了工业平稳运行。全市重点企业及新上大项目达产达效拉动全市工业经济增长15个百分点以上。大力推进对俄合作适应性战略调整，有效地遏制了进出口下滑势头。坚持采取科技抗旱与设施抗旱相结合，农业生产实现灾年大丰收。一年来，我们上下一心，同舟共济渡难关，始终保持强大的发展合力。在国家、省委省政府和省直各部门的大力支持下，市委统揽全局，政府、企业、市民并肩携手，共克时艰，形成了全市上下一盘棋，共渡难关保增长的强大合力。市领导亲自带队，各战线分兵把口，数百人次跑省进京，共为381个项目争取国省扩大内需资金8.7亿元，累计争取资金达14.4亿元。多年来坚持不懈地抓项目建设促经济发展，积攒的后劲在应对危机的过程中得以显现。

　　各位代表，金融危机没有阻挡住佳木斯前进的脚步，经过危机的考验，我们的后发优势和发展势能加速释放，产业素质和发展基础更加坚实，发展后劲和增长亮点不断显现，昭示着佳木斯跨越发展的美好前景。在此，我代表市政府，向全市人民，向所有关心、支持佳木斯发展的各界朋友，致以崇高的敬意和衷心的感谢！

　　过去的一年，我们取得了来之不易的成绩，但更要清醒地看到差距和问题，经济增长的内在动力依然不足，科技创新对经济发展的贡献率还不高，非公有制经济比重比较低；产业结构亟待优化，工业总量小，对财政的贡献率低，县域经济发展速度不够快，实力还不强；财政收支平衡的压力大，收入主体税源规模小，可持续增长的税源不多，扩大内需项目配套资金缺口较大，民生改善所需投入呈刚性增长态势，到期债务还债压力较大；发展环境无论是硬件还是软件都还需要改进和完善，城市建设、管理、经营等方面尚需进一步加强；工作作风有待进一步转变，政府的服务质量和效率需要进一步提高，部门利益倾向需要进一步克服，一些政府工作人员的公仆意识需要进一步增强。对于这些问题，我们一定高度重视，认真加以解决。

　　二、2010年主要工作

　　2010年，是我市“十一五”攻坚收官、“十二五”规划奠基的关键之年。纵观国际国内形势，国内经济企稳回升态势基本形成，世界经济呈现复苏迹象，佳木斯的发展正处于新的机遇期。一是中央经济工作会议决定继续实施积极的财政政策和适度宽松的货币政策，扩大内需保持经济平稳较快增长，我们加快发展的大环境仍然有利。二是中央提出把调整结构、转变发展方式作为今后经济工作的重大方针和战略任务，使佳木斯的发展战略与国家政策导向的契合度更高，为我们发挥特有的资源环境和产业基础优势，加快调整经济结构，走新型工业化道路，大力发展战略性新兴产业、低碳经济带来难得的历史机遇。三是中央要求黑龙江发展现代化大农业，将有利于我们发挥发展现代农业的显著优势，极大地促进我市农业“五化”建设，推动我市现代农业示范核心区进程，在发展现代化大农业上独树一帜，大有作为。四是胡锦涛总书记提出要求黑龙江要成为沿边开放的重要桥头堡和枢纽站，中俄两国正式签署了《中国东北地区老工业基地与俄罗斯远东地区合作规划纲要》和《中俄投资合作规划》，对发挥我市的地缘、口岸通道、对俄合作基础优势，特别是黑瞎子岛开放开发、同江中俄铁路大桥建设的重大合作题材优势，打造沿江开放带十分有利。五是国家提出加快中小城市发展和城镇化步伐，推进城乡一体化的战略，将有利于我们发挥中心城市优势，加快区域中心城市建设，提升城市功能和辐射带动力，引领新农村建设，加快我市城乡一体化进程，形成新的经济增长点。

　　面对新的形势和全市人民的期盼，我们将认真落实省委经济工作会议和市委十一届七次全会精神，深入贯彻科学发展观，加快发展方式转变，深入推进“两区两带一城”建设，全力实施“十项重点工程”，强基础、调结构、提效益、惠民生、促和谐，努力实现经济社会科学跨越发展新突破！

　　主要预期目标是：确保地区生产总值增长13%，规模以上工业增加值增长16%，全社会固定资产投资增长35%以上，社会消费品零售总额增长15%，外贸进出口总额增长15%，财政一般预算收入增长15%，万元GDP综合耗能下降4.7%，化学需氧量排放量下降2.6%，二氧化硫排放量下降0.4%，城镇人口登记失业率控制在4.5%以内，人口自然增长率控制在5‰以内。城镇居民人均可支配收入增长8%，农民人均纯收入增长6%以上。

　　为此，我们将重点做好以下工作：

　　(一)坚持推进大项目建设，增强发展后劲，形成保增长、调结构的强力支撑。以60个亿元以上项目为重点，坚持推进大项目建设，全年固定资产投资突破200亿元，达到230亿元以上。一是加快基础设施项目建设。在公路方面，加快建设集同高速公路、富锦松花江公路大桥、抚远至乌苏镇二级公路等在建项目建设，开工建设黑瞎子岛上岛桥、汤原至依兰界二级公路。建设农村公路600公里，实现所有的乡镇和行政村都通上水泥路的目标。在航运方面，加快建设抚远深水港和富锦东平港区。在航空方面，开工建设抚远支线机场。在铁路方面，加快前抚铁路建设，争取开工建设同江中俄跨江铁路大桥、哈尔滨至佳木斯铁路客运专线。在水利方面，加快临江、幸福、悦来、引汤等灌区建设，力争再开工建设一到两个灌区，开工建设黑瞎子岛国土护岸工程。二是加快重点产业项目建设。计划开复工重点产业项目35个。全市工业重点推进37个投资额超5000万元以上项目，其中投资超亿元项目29个，投资55亿元，投达产项目20个。七彩毯业年产1000万条高级拉舍尔毛毯等10个重点项目开工建设。三和亚麻年产770万米高支高密精纺亚麻细布、龙江福年产13.5万吨纸浆改造等18个续建项目，尽快投产达效。开复工农机大市场等商贸流通项目9项，投资16亿元。三是加快重大项目前期工作。重点做好佳木斯航电枢纽、佳木斯机场迁建、佳木斯西北绕城高速公路及跨江公路大桥、佳双同和佳鹤铁路提速扩能改造、同江至抚远高速公路、富锦百万亩松花江灌区、佳铝电解铝异地改造、中广核核电、鲁能煤电化等项目的前期工作。四是进一步加大埠内外融资力度。加快引进龙江银行等外埠金融机构，积极组建地方合作银行和村镇银行，组建小型金融机构，建设金融服务大厦，发行城投公司债券10亿元。改善金融生态环境，创新金融产品和服务，深化政银企合作，力争贷款余额增幅保持在30%以上。支持电机股份、沃尔德电缆等成长性好的企业尽快上市融资。创新招商引资方式，以优势产业为依托，以行业龙头企业为主攻目标，推进产业链招商，掀起新一轮招商热潮，全年国内招商引资到位资金实现120亿元，增长30%；实际利用外资实现5600万美元，增长10%。

　　(二)优化工业经济结构，大力发展战略性新兴产业，培育新的增长点，实现工业总量三年翻番。全市工业实现主营业务收入250亿元，增长20%以上，确保完成工业总量三年翻番目标；实现利税20亿元，增长10%以上。一是确保重点骨干企业快速发展。加强经济运行监控与协调服务，对主营业务收入亿元以上的47户企业实行重点包保，促其满负荷生产。促进电机、煤机、迪尔佳联、北方水泥、沃尔德电缆、益海粮油、雨润食品、东兴煤化工、佳联收获等企业新建改造项目达产达效，增加新的增长点。二是加大结构调整力度。加快发展以低碳为特征的新能源、新材料、生物和先进装备制造四大战略性新兴产业。新能源产业重点开工建设同江华能风电、富锦五顶山风电、桦南龙源黄团岭风电、汤原渠首风电、桦川宝山风电等5个项目，确保新增装机能力20万千瓦，总装机突破80万千瓦，力争达到100万千瓦，继续保持全省风电领先。新材料产业重点加快海绵钛项目建设，通过与中国建材等企业合作，加快高标号水泥、商品混凝土、功能玻璃、环保板材等项目开发建设。北方水泥日产5000吨新型干法水泥熟料生产线及余热发电项目建成投产；佳星玻璃日熔600吨在线镀膜玻璃项目开工建设。生物产业重点推动益海嘉里30万吨油脂，南华日处理6000吨甜菜，中粮、天稻、付士等90万吨稻米深加工项目开工建设，打造“东北大米之都”；开工葵花药业、多多药业等改造项目，建设现代中药产业基地；推动秸秆发电、稻壳发电等生物质能项目建设。先进装备制造业重点推进迪尔佳联年产20000台联合收割机、常发集团联合收割机和大马力拖拉机、骥驰和龙江大中马力拖拉机、省农机公司农机具项目，加快“东北农机城”建设步伐；佳电股份核电电机实现投产。传统产业改造提升重点实施龙江福30万吨纸浆、东兴煤化工三期甲醇等项目。三是大力发展县区工业。制定和实施加快县域经济、城区经济发展的总体规划和政策意见。实施县区工业提速工程，壮大县区工业规模，打造一批资源增值型、科技型、外向型、服务型立县立区支柱企业，县域规模以上工业增加值增长20%以上，财政收入增长17%以上。城区规模以上工业增加值增长20%以上，财政收入增长15%以上。

　　(三)加快发展现代化大农业，深入推进现代农业综合开发试验核心区建设，全面提升新农村建设水平。一是加快粮食产能工程建设。实施“一带三区”优势种植产业带规划，即以汤原、郊区、桦川、富锦佳抚路两侧为主的水稻生产带；以抚远、同江、富锦为主的大豆生产区；以佳木斯南郊、桦南为主的玉米生产区；以各县(市)区独具特色的经济作物为主的特色作物生产区。积极调整种植结构，压缩大豆面积，增加种植水稻面积80万亩，达到420万亩；增加玉米种植面积40万亩，达到440万亩，保持良好的粮食增产势头，全年粮食总产稳定在90亿斤以上，力争突破100亿斤。加快农业开发高标准农田建设，打造水稻高标准农田示范带3个，即市区东出口到桦川星火乡、汤原太平川、桦南大鲜各10万亩。在桦南、汤原、抚远县各建设1个投资1000万元以上的农机合作社；各县(市)区完成购置80马力以上拖拉机200台。加速推进规模化生产，新增规模化种植面积300万亩，总量突破1000万亩。深化场县合作，农垦代耕面积达到210万亩。二是大力发展畜牧业。围绕出栏392万头生猪、新增1万头奶牛等发展目标，重点抓好20个标准化规模养殖示范场(小区)，全市规模化养殖场增加50个，达到2121个。牧业产值实现56亿元以上，增长11.6%，占农业总产值比重达到47.5%，牧业人均纯收入增加100元。加快发展特色水产养殖，全市养鱼水面发展到26.5万亩，水产品产量4.5万吨，渔业经济总产值实现10.9亿元，占农业总产值的10%。切实抓好禽流感、口蹄疫等动物疫情的防控工作，力保无重大疫情发生。三是坚持不懈推进农业产业化。继续推进水稻、大豆、玉米、甜菜、生猪等优势农畜产品“百万级”深加工产业群建设，全力扶持中储粮、中粮、北大荒、吉庆、付士、天粮、益海嘉里、九阳、南华、雨润、金锣、四海、光明、阳光等大型农业产业化龙头企业经营好已投产的项目，建设好新建项目，并整合小型加工企业，培育地方品牌，形成产业集团，建设中国绿色食品大市名市。四是拓宽农业增收渠道。以边境口岸地区为重点，建立对俄蔬菜出口专业村(屯)和示范园区，形成对俄果菜出口优势的产业带；扩大对俄农业合作，新增种植面积12.14万亩，达到86.88万亩。加大农村劳动力转移力度，新增转移人数3万人。新增植树造林面积34.5万亩。

　　(四)扩大对外开放，培育消费热点，发展现代服务业，确保第三产业持续快速增长。一是推进对俄经贸合作适应性战略调整。适应俄罗斯政策调整，推动对俄经贸合作方向和领域由以贸易为主向贸易与投资合作并重转变。组织企业先选优选合作项目，重点实施钛、铁、镁、铝、煤炭、木材、钻石等资源开发项目合作。实施“引大联强”战略，积极引进一批有实力的企业到俄罗斯投资开发和贸易合作。全力办好首届中俄(佳木斯)农机展销洽谈会。开通佳木斯至哈巴罗夫斯克等国际航线。二是加快对俄合作园区建设。高起点做好黑瞎子岛对俄合作示范区的国际招标规划设计，积极争取国家特殊政策支持，高水平搞好上岛桥等基础设施项目建设，积极与俄地方政府沟通衔接，加快双方合作规划开发进程。对俄合作产业园区重点抓好同江、抚远、富锦木材加工园区、同江下列木材加工园区建设，支持永安集团在俄比罗比詹市、共青城市，抚远兴大贸易公司在哈巴罗夫斯克市的木材加工项目建设，力争尽快投产达效。培育壮大境外农业园区。规划建设佳木斯、富锦、同江、抚远等4个国际物流园区。三是大力推进市场体系建设。加快推进农机大市场扩建、商业大厦改造、秋林公司改建、红旗大市场二期建设等项目续建工作。推动家乐福超市、沃尔玛超市、凯联家居博览中心、千里汽车城、农产品综合交易中心等一批重点项目开工建设。推进三江温州批发城三期扩建和西区商业综合体项目，争取开工建设。深入推进“家电下乡”、“万村千乡市场”工程和“双百市场”工程，进一步扩大农村消费服务，构建科学合理、流通快捷的农村消费品流通网络。四是推进全国优秀旅游城市建设。精心办好四个重大节会。加快推进三江特色旅游带及重点景区项目建设，启动黑瞎子岛省级旅游名镇和7个市级旅游名镇建设，建成敖其湾赫哲文化旅游区，打造一批精品旅游景区和名牌旅游产品。全年预计接待海外旅游者13.5万人次，创汇2430万美元，增长10%；预计接待国内旅游者232万人次，旅游收入9.5亿元人民币，增长15%。

　　(五)推进创新型城市建设，提升自主创新能力，加快高新技术园区建设，促进高新技术产业发展。一是加快高新技术产业开发区建设。尽快完成高新区建设和产业总体规划，强力推进农机产业园，细化钛产业、新型建材、医药等产业园区规划并加快实施进度，集中摆放产业项目，配套建设基础设施，尽快形成聚集效应。围绕生物医药、机电一体化、新材料等高新技术领域开展科技合作招商引企，使之成为我市承接高新技术项目，转化高新技术成果，实现高新技术产业化的重要基地。二是加快农业科技示范园区建设。重点发挥和依托省农垦科学院、省农科院佳木斯分院和省农业职业学院的人才和技术聚集的优势，打造三江平原最大的现代农业科技示范区——佳南农业科技园区，筹资2500万元建设农博馆、智能化温室等标志性项目，扩大园区规模，争取成为国家级农业科技园区。发挥农业科技园区创新与示范功能，促进农业科技成果的应用与转化，辐射和带动全市农业科技示范园区建设上水平。三是打造科技创新平台。大力支持农机、医药、电缆等在全国或区域处于领先水平的省级工程(技术)研究中心建设，力争电机股份建成国家级工程技术研究中心。建成谷物联合收割机、钛合金两个国家级质量技术检测中心。以提升市政府与佳木斯大学合作的中俄科技合作中心功能为重点，扩大对外技术合作领域。加快佳木斯大学城和大学科技园建设，使之成为高层次人才培养中心、创新人才集聚高地和高技术研发基地。四是强化创新体系建设。引导建立产业技术创新战略联盟，推动政产学研金介的有机结合，增强优势产业核心竞争力。实施专利试点工程，培育一批国家和省级重点新产品；力争完成电机股份博士后科研工作站建站工作，培植建站后备梯队2-3家。

　　(六)增强经济发展动力，大力发展非公有制经济，推进重点领域和关键环节改革。一是促进民营经济全面发展。抓住国家级创业型城市试点的机遇，用足国务院和省政府关于鼓励支持非公有制经济和中小企业发展的政策，实施“育小、扶中、抓大”战略、中小企业成长工程、创业示范工程、“小伙伴”工程。加强企业信用体系建设，解决融资难等各种难题，确保各项政策措施落实到位。市、县两级创业孵化园孵化创业示范企业100个，创建创业型社区30个，创业型村屯30个，培育选树创业小老板3000人。全年新增规模企业40户，年经营额超亿元企业增加到70户，新增6户；千万元以上企业增加到550户，新增44户。全市民营经济实现增加值93亿元，增长25.7%。二是继续深化国有企业改革。加快推进佳西热电、佳星玻璃、鹿灵制药、侨龙毛毯、佳凤啤酒、东方市政、燃气公司、供水公司等企业的改制工作，实现招商、改制、职工安置同步推进。做好改制企业收尾工作，解决职工养老、医疗、失业保险接续、人事和财务档案管理、离休等相关人员管理和国企改革留守中心的组建与管理等问题。三是加快农村综合改革。扩大农村土地流转规模，新增20个整村流转村，积极推动整乡土地流转。探索国有林权制度改革有效形式，建立3-5个国有林权制度改革联户经营组织，抓好3个国有林场改革。创新农村经营组织形式，新发展农民专业合作社40个，培养示范社20个。组建3家村镇银行，做好同江市农村合作银行筹建工作，扩大小额贷款公司规模，加快组建市级农村信用社统一法人社。四是扎实推进政府机构和事业单位改革。年内完成市、县(区)政府机构改革，积极稳妥地推进事业单位改革，成熟一批改革一批，切实解决政事分开、政资分开、事企分开、政府和事业与社会中介组织分开问题。加快推进教育和医疗卫生制度改革。

　　(七)加快城镇化和城乡一体化进程，加大城市建设的投入改造力度，提升经营管理水平，实现城市环境大变样。一是加快城镇化建设。高起点编制全市城镇体系规划，采取创新突破的政策措施，加速城镇化和城乡一体化进程。二是加快推进滨水城市建设。依托我市沿江滨水的特有优势，大力实施滨水景观建设。高起点完成佳木斯市区、汤原、桦川、富锦、同江、抚远等滨水城镇建设规划，并启动实施一批重大标志性项目建设。进一步提升佳木斯市区松花江沿线景观带建设水平，续建新建沿江公园二、三期，十里沿江景观带全线建成。规划建设江北沿江湿地公园。坚持高标准规划设计，巩固提升水源山、四丰湖等景区休闲旅游功能，加快柳树岛的规划开发。引进国内外顶尖的开发企业鲁能置业集团、益海嘉里集团投资建设省内一流的鲁能滨江新城、香格里拉滨江商住中心。规划启动伟业“低碳生态城”项目建设，打造生态环保顶级小区。对英格吐河、音达木河、王三五河、杏林河等四条城市内河综合整治进行统一规划，加快启动建设。三是全面实施城市四大出口全线整体配套改造建设。对北、西、南、东四个出口和滨江路的道路及沿线的棚户区、建筑物、基础设施进行全线整体改造升级。横向贯通胜利路、长安路、光复路三条东西交通大动脉，纵向打通断头路，新建科技大道、兴国大道等道路16条，拓宽改造安庆街、解放路等道路15条，维修长安、友谊等道路10条，新建和改造英格吐河桥、光明桥等桥梁15座，新增停车位1万个，全面提升道路交通承载力。确保省运会场馆及周边改造工程7月底前全部完工。四是加快城市“三供两治”项目建设。加快推进清华同方与自来水公司TOT项目合作，提高城市供水能力和服务水平。支持法国达尔凯公司BOT供热项目，加快城市热管网改造升级，尽快完成国电佳西2×30万千瓦热电联产新建和华电“七期”扩建立项工作，争取开工建设，扩大集中供热面积。全年分户改造面积30万平方米，新开发楼房上热面积160万平方米，分散锅炉房并网50万平方米。加快推进大庆方兴公司启动江北钻打新气井方案，争取开工中煤能源达连河至佳木斯天然气长输管线，天然气用户达到7.1万户，管道天然气普及率达到60%。五是全面实施绿化美化亮化工程。实施城市绿化“85431”工程，即绿化长安西路等8条主要道路与绿岛；绿云公园等5个公园；北出口等4个出口；杏林河等3条河旁绿带；新建1个苗木生产基地。完成长安路、和平路等5条重点街道楼体及39个路口楼体亮化改造。对现有五个广场进行主题特色改造，进一步加强花园式小区建设。六是全力推进棚户区改造工程。安排改造项目45个、220万平方米，确保完成三年主城区棚户区改造任务。改造67个、40万平方米旧小区。七是加大环境综合整治力度。继续推进“三优共建”，全面实施垃圾源头治理工程、市容市貌整顿升级工程和楼体美化工程。依法加强城市管理，大力整顿交通秩序，实现管理升级上水平。强力推进松花江流域水污染防治，在建项目全面完成。推广和应用节能技术、节能装备，建设节能建筑，全面完成“十一五”节能减排目标。

　　(八)全力保障和改善民生，维护社会稳定，促进社会和谐，加快提高城乡居民的生活质量。一是统筹城乡就业。全年实现城乡新就业13.1万人，城镇新就业8万人，下岗失业人员再就业6.5万人，城镇登记失业率控制在4.5%以内。加强劳动技能培训，完成再就业培训2.6万人，创业培训1万人，农村劳动力转移培训2万人，企业职工在职培训1万人，培养高技能人才1500人。二是加快完善社会保障体系。新农保试点范围内农民参保率达到70%，60周岁以上人口享受基础养老金达到100%；继续加大各项社会保险的扩面力度，城镇社会养老保险、职工基本医疗保险和城镇居民医疗保险在制度覆盖范围内，基本实现应保尽保。努力解决困难群众住房问题，新建实物配租4886套，新增租赁补贴1324户，达到14361户。进一步完善社会救助体系，加快福利项目建设，推进敬老院建设，提高各种福利机构的服务水平，推进社区养老、居家养老工作深入发展。推进城乡社区建设提档升级，开展和谐社区、农村社区试点工作，更好地发挥基层管理服务平台的作用。续续实施开发式扶贫，整村推进93个村实现整体脱贫。严格兑现国务院关于提高城镇企业退休人员养老保险待遇的决定，确保人均提高10%以上，继续提高机关事业单位人员津贴补贴，住房公积金缴存比例提高2个百分点。三是加快社会事业发展。全面推进素质教育，加快标准化学校建设，促进教育均衡、公平发展。加快普及高中教育，提升职业教育水平。图书馆、广电大厦竣工使用，博物馆新馆争取开工建设。建立基本药物制度，健全城乡基层医疗卫生服务体系，促进基本公共卫生服务逐步均等化，实现“新农合”参合率98%，城市社区卫生服务人口覆盖率88%。推进计划生育惠家工程，解决城市和城镇无业居民独生子女父母奖励费，各级财政确保计划生育事业费达到国家规定的人均30元标准。四是举办一届高水平的省运会。举全市之力，把第十二届省运会办成一届精彩圆满、成功难忘的省运会，承办好第八届省少数民族运动会。促进全民健身条例贯彻落实，提高全市群众体育和竞技体育水平。五是加强和谐社会建设。进一步加强社会治安综合治理，深入实施“平安佳木斯”创建活动，严格落实维稳工作责任制和稳定风险评估制度。着力提升维稳能力、打击能力、防控能力，全力确保全市社会安定。继续深入开展县(市)区领导大接访、干部大下访和“三访”专项治理活动，着力解决群众切身利益问题。完善群体性事件预防处置预案和矛盾纠纷调处机制，及时化解社会矛盾。全面落实企业的生产主体责任和政府的监管主体责任，打好安全生产隐患治理攻坚战，强化应急救援体系建设，坚决杜绝发生重特大事故。

　　(九)加强政府自身建设，提升政府执政能力，提高公务员素质，塑造人民更为满意的政府。以践行科学发展观为统领，不断解放思想，与时俱进，进一步提高推动科学发展和社会和谐的能力，着力打造学习型、创新型、效能型和服务型政府。一是进一步提高政府行政效能。自觉接受人大、政协监督，人大代表建议和议案、政协委员提案办结率达到100%。建立健全抓落实的领导机制和工作机制，首问负责、限时办结、责任追究等“六项制度”在各级政府及部门的覆盖率要达到100%，做到事有专管之人，时有限定之期，标有奖惩之措，真正把决策部署落实到具体措施上、落实到最终成效上。强化应急管理，提升公共突发事件预警和处置能力。新建行政服务中心大厅，创建良好的服务环境。二是切实转变工作作风。各级政府及政府各部门要切实增强责任感、使命感和危机感，紧紧扭住发展这个第一要务，集中精力抓推进、抓落实，多干事、干成事。坚持深入基层，深入实际，解决问题。始终把群众的利益放在首位，满怀激情创建美好家园，心怀真情解决群众疾苦，挥洒豪情推动城市发展。三是加强廉政建设。坚持廉洁从政、从严治政，按照“标本兼治、综合治理、惩防并举、注重预防”的总要求，严格执行党风廉政建设各项规定，严肃查处商业贿赂案件，推动建筑工程领域突出问题专项治理，严厉打击各类违法乱纪行为，在全市上下营造风正心齐谋发展的浓厚氛围。

　　各位代表，佳木斯已站在加速发展的新起点上。面对251万人民的期盼和重托，市政府决心在市委的领导下，带领全市人民戮力同心，奋发图强，为全面超额完成“十一五”规划目标，实现全市经济社会科学跨越发展而努力奋斗！
